# Clear Cell Renal Cell Carcinoma With Brain Metastases Treated With Complementary Ketogenic Metabolic Therapy: A Case Report

**DOI:** 10.7759/cureus.84962

**Published:** 2025-05-28

**Authors:** Alfredo Acevedo, Martin Zapata Laguado

**Affiliations:** 1 Clinical Oncology, Universidad El Bosque, Bogota, COL; 2 Clinical Oncology, Instituto Nacional de Cancerología, Bogota, COL

**Keywords:** aerobic glycolysis, brain metastasis, immunotherapy, ketogenic metabolic therapy, kidney cancer

## Abstract

A 65-year-old woman was first diagnosed in 2011 with stage III clear cell renal cell carcinoma (pT3aNxM0) of the left kidney, managed successfully with radical nephrectomy. She remained disease-free for three years. In 2014, she developed a metachronous stage III papillary thyroid carcinoma (T1N1M0), treated with total thyroidectomy, nodal dissection, and adjuvant radioactive iodine therapy (100 mCi). In 2019, two pulmonary lesions were detected in the left upper lobe. Biopsy confirmed metastatic clear cell carcinoma. Due to intolerance to sunitinib, the patient was treated with pazopanib. By September 2022, she developed four intracranial metastatic lesions, predominantly in the right frontal lobe and bilateral mesial temporal lobes. A neurosurgeon performed a biopsy, confirming metastatic clear cell carcinoma. The patient underwent whole-brain radiotherapy over five sessions and began treatment with nivolumab. After receiving five cycles of immunotherapy, the patient experienced a seizure associated with edema around the dominant lesion and neurological decline. She was treated with anticonvulsants and a short steroid course, leading to functional recovery. We used this treatment for around 10 days and continued immunotherapy and anticonvulsant therapy. In January 2023, ketogenic metabolic therapy (KMT) was initiated using a 3:1 ratio ketogenic supplement (KetoVie). Ketone levels and neurological status were closely monitored. Upon confirmation of ketones in the urine or blood, therapy adjustments were made to optimize adherence. By July 2023, the patient no longer required assistance for medical appointments, discontinued anticonvulsant therapy, and retained full cognitive function. In January 2024, the patient maintained a partial response, and dietary supplementation was stopped. This case highlights the potential role of KMT as a safe and complementary approach in advanced clear cell renal cell carcinoma. KMT may enhance the efficacy of immunotherapy and radiotherapy, contributing to improved progression-free survival and neurological function without increasing toxicity.

## Introduction

Kidney cell carcinoma is the 14th most common cancer worldwide. The age-standardized mortality rate (ASR) is approximately 12.8 per 100,000 in the USA and 3.8 per 100,000 in Colombia [1]. Metastatic disease accounts for 11% of cases and is associated with a poorer prognosis compared to localized or locally advanced disease. Central nervous system (CNS) metastases occur in 8% of patients and are linked to a worse prognosis than metastases at other sites [2].

Phase III clinical trials often exclude patients with active CNS metastases, resulting in limited evidence to guide the management of this population [3-7]. The CheckMate 920 trial is a notable exception, a phase III study specifically designed to evaluate the safety profile of nivolumab and ipilimumab in patients typically excluded from other trials, such as those with brain metastases. In a cohort of patients with CNS involvement (N = 28), the objective response rate was 32%, with a median response duration of 24.0 months and no new safety concerns identified [8]. When feasible, local treatment with surgery or radiation remains the standard of care, particularly for patients presenting with symptoms.

It is important to emphasize that the current standard of care for patients with metastatic clear cell renal cell carcinoma involves immunotherapy in combination with other agents, including either a combination of anti-CTLA-4 and anti-PD-1 therapies or a combination of anti-PD-1 therapy with a tyrosine kinase inhibitor (TKI) as the main recommendation. However, some patients started with a TKI mono-agent.

Following first-line therapy, treatment sequencing can be challenging. In general, switching to a different TKI is recommended for patients previously exposed to this class, while those not treated with TKIs in the first line may begin TKI therapy in the second line. Another drug that is an emerging agent in this therapeutic landscape is belzutifan, which has shown promise in early studies, and the genitourinary (GU) oncology community is currently awaiting more definitive data from ongoing trials.

Despite advancements in systemic therapies, a significant proportion of patients with metastatic clear cell renal cell carcinoma experience poor outcomes. For this reason, it is crucial to explore complementary therapeutic strategies, including dietary interventions, in this patient population.

Glucose and glutamine are the primary energy sources for proliferating tumor cells. These cells often undergo metabolic reprogramming to favor aerobic glycolysis, a phenomenon known as the Warburg effect, which supports their survival and proliferative capacity. Targeting this metabolic vulnerability by inhibiting glycolysis through increased circulating ketone bodies offers a novel therapeutic strategy. The ketogenic diet, a high-fat, low-carbohydrate dietary regimen, is designed to reduce glucose availability while increasing ketone production. A commonly used macronutrient ratio in this context is 3:1 (fat-to-carbohydrates plus protein) [9]. The antitumor and antiangiogenic effects of KMT were first demonstrated in mouse models of astrocytoma and later extended to human models of glioblastoma [10,11]. In recent years, the application of KMT has expanded to include other types of tumors.

In this report, we share our experience with a patient who had brain metastasis secondary to clear cell renal cell carcinoma, treated with a combination of TKI, immunotherapy, and KMT, yielding outstanding results.

This article was previously presented as an abstract at the 19th European Association of Neuro-Oncology Meeting 2024.

## Case presentation

**Figure 1:**
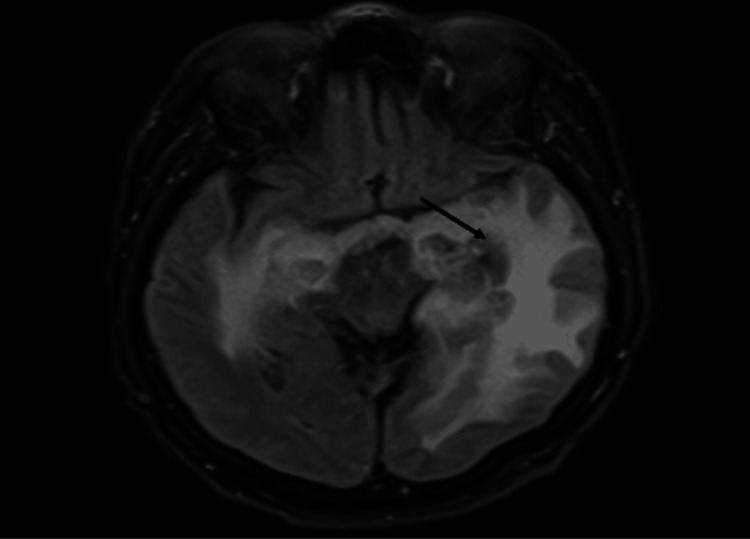
MRI from September 2022 showing edema in the mesial regions of both temporal lobes, with significant involvement of the hippocampi and periventricular distribution in left temporal lobe

**Figure 2:**
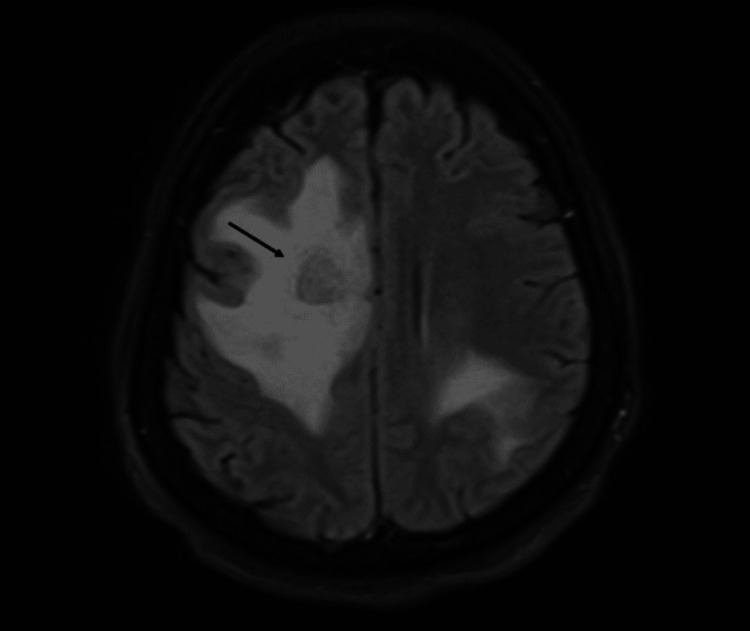
MRI from September 2022 showing intracranial metastases with intra-axial cortico-subcortical localization and supratentorial distribution in both cerebral hemispheres. Extensive vasogenic edema is present, predominantly affecting the right frontal lobe

A 65-year-old woman was first diagnosed in 2011 with stage III clear cell renal cell carcinoma of the left kidney (pT3aNxM0), which was managed with radical nephrectomy. After remaining disease-free for three years, she developed metachronous stage III papillary thyroid carcinoma in 2014 (T1N1M0). This was managed with total thyroidectomy, nodal dissection, and adjuvant iodine therapy (100 mCi). In 2019, two new lesions were identified in the left lung upper lobe (measuring 16.5 mm and 11 mm). A biopsy following wedge resection of the larger lesion confirmed the recurrence of clear cell carcinoma. She began treatment with sunitinib but discontinued after one cycle due to poor gastrointestinal tolerance. The treatment was switched to pazopanib, which she tolerated well, and her lung disease remained stable. However, in September 2022, imaging revealed four brain lesions, two in each cerebral hemisphere, mainly involving the right frontal lobe and the mesial regions of both temporal lobes (Figure 1 and Figure 2). A biopsy performed by a neurosurgeon confirmed metastatic clear cell carcinoma.

**Figure 3:**
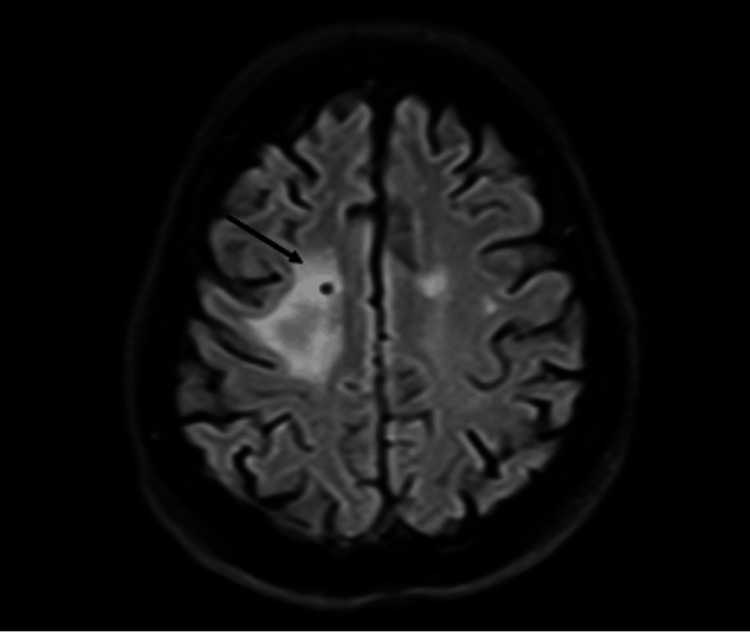
MRI from January 2024 showing findings consistent with a partial response of hemorrhagic supratentorial intra-axial metastatic disease

**Figure 4:**
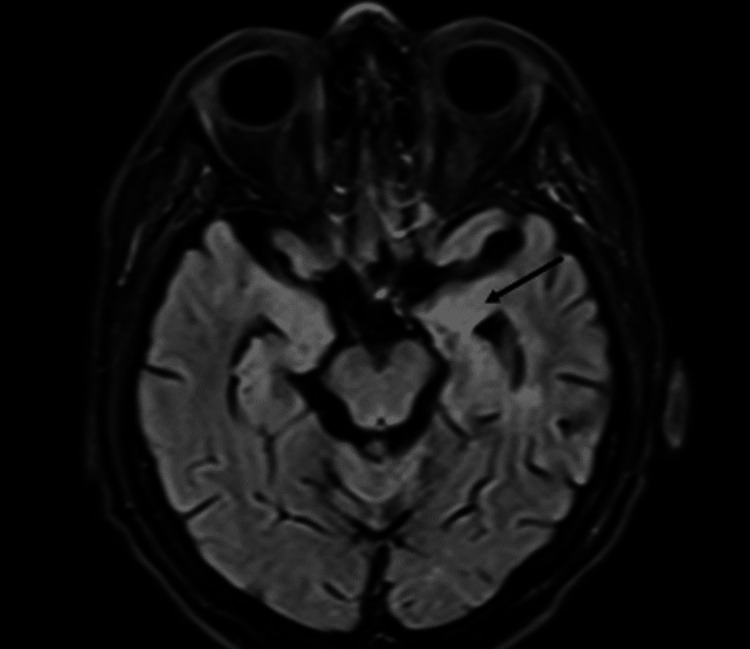
MRI from January 2024 showing the mesial region of the hippocampus with significant volume reduction and near-complete resolution of adjacent vasogenic edema compared to the previous study

The patient received intensity-modulated radiation therapy and whole-brain radiotherapy (20 GY over five daily sessions). Based on the CheckMate 025 trial, treatment with nivolumab was initiated in September 2022. After receiving five cycles of immunotherapy, the patient presented in January 2023 with a new-onset seizure. Imaging showed edema at the site of the primary lesion and neurologic worsening, although the disease remained stable. She was treated with anticonvulsants and a short course of steroids for around 10 days, which led to functional recovery. Immunotherapy and anticonvulsant therapy were continued. In the same month, KMT was introduced at a 3:1 fat-to-carbohydrate ratio. Ketone levels and neurological status were closely monitored. Dietary adjustments were made to support adherence after confirming ketone presence in urine or blood. By July 2023, the patient no longer needed assistance for medical appointments, discontinued anticonvulsants, and showed preserved cognitive function. Radiological imaging showed a partial response in both the CNS and lungs. By January 2024, after 14 cycles of nivolumab, the partial response persisted (Figure 3 and Figure 4), and dietary supplementation (KetoVie) was discontinued. The patient continued with a low-carbohydrate diet and remained fully independent in daily activities, with no cognitive deficits.

## Discussion

The current evidence on KMT comes from observational data, three prospective cohort studies, and several case series [12]. Most cases involve gliomas, but there are also reports of unresectable or metastatic lung, pancreas, colon, thyroid, ovarian, breast, parotid, endometrial, and stomach cancers, as well as melanoma and osteosarcoma [13].

Among these case series, only one previously documented a patient with clear cell renal carcinoma [14]. Our patient has remained on KMT for 15 months, marking the longest reported use of this strategy for this cancer type. The sustained partial response observed may be due to the combined effect of immunotherapy and KMT.

Potential side effects of KMT include weight loss, dyslipidemia, hyperuricemia, kidney damage, and liver enzyme abnormalities. However, none of these were observed in our patient.

KMT has been linked to alterations in the tumor microenvironment, including hypoxia, inflammation, angiogenesis, and vascular permeability. However, its exact impact on tumor immunity remains unclear. In mouse models of glioblastoma, KMT has been shown to reduce NF-kB activation and cyclooxygenase-2 expression, both of which are involved in hypoxia-induced immunosuppression. Based on these findings, it is hypothesized that KMT may help enhance antitumor immune responses. Regulatory T cells, which produce IL-10, contribute to immune suppression within the tumor microenvironment. CTLA-4 and PD-1, which are found on the surface of activated effector T cells, serve as key immune checkpoints. Animal studies suggest that KMT may reduce the expression of PD-1, CTLA-4, and their ligands on tumor-infiltrating cells. This may explain the observed synergistic effect of combining KMT with immunotherapy [15].

At MD Anderson Cancer Center, Richard et al. analyzed in vitro models and preclinical murine tumor models to understand the impact of KMT on renal cancer cell proliferation [16]. They obtained kidney specimens from 18 patients and successfully cultured clear cell renal cell carcinoma from eight of them. The cells were then exposed to KMT (using 5 mM of acetoacetate and beta-hydroxybutyrate) in a low-glucose environment. When compared to cells grown in high-glucose conditions, there was a significant 83% reduction in cell viability with the KMT approach. To evaluate the effect of KMT in living organisms, renal cancer cells were implanted into syngeneic mice, which were then treated either with ketone bodies or anti-PD-1 immunotherapy. Tumor volume was significantly reduced in these mice. This may be because KMT alters fatty acid and glutamine metabolism, which increases PD-L1 expression, potentially enhancing the immune response and supporting synergy with immunotherapy [16].

In line with this, the KETOREIN trial at Gustave Roussy Institute aimed to assess the response rate (partial or complete response) to a combination of nivolumab and ipilimumab concomitant to a special diet (ketogenic, continuous, intermittent, or standard diet). However, the trial was terminated early due to recruitment difficulties [17].

Another study, the CETOREIN trial at Angers University Hospital, is investigating the safety and tolerability of a ketogenic diet combined with standard treatments in patients with metastatic renal cell carcinoma. Recruitment has been completed, and results are pending [18].

## Conclusions

This case supports the use of KMT in clear cell renal cell carcinoma, where it may work synergistically with TKIs, immunotherapy, and radiotherapy. The combination has the potential to improve progression-free survival and functional outcomes without increasing toxicity. These promising results highlight the need for further studies to explore the role of KMT as a complementary treatment option in similar patient populations.
